# Ultrasound Measurement of Cervical Length at 36-36+6 Weeks’ Gestational Age to Predict Spontaneous Onset of Labor and Mode of Delivery

**DOI:** 10.7759/cureus.108153

**Published:** 2026-05-02

**Authors:** Arundhathi Ramadas, Bindu Menon, Aneesh Mangalasseril, Ramadas Changerath

**Affiliations:** 1 Obstetrics and Gynecology, Jubilee Mission Medical College and Research Institute, Thrissur, IND; 2 Radiology, Jubilee Mission Medical College and Research Institute, Thrissur, IND; 3 Institutional Research, Khalifa University, Abu Dhabi, ARE

**Keywords:** bishop’s score, cervical length, labor onset biomarkers, maternal-fetal medicine, obstetric assessment, pre-induction cervical length, preterm birth risk, spontaneous onset of labor, transvaginal ultrasound

## Abstract

Background and objective

Cesarean section rates are rising globally, including in India. Following the ARRIVE trial, elective induction at 39 weeks has become more common. Cervical length is included in the Bishop score for assessing cervical favorability; however, the Bishop score is subjective and has limited predictive value. This study proposes that an objective third-trimester transvaginal ultrasound measurement of cervical length at 36 weeks may better predict spontaneous labor onset and guide delivery management. The objective of this study is to determine the correlation between cervical length at 36-36+6 weeks and spontaneous labor onset and to assess its effectiveness in predicting mode of delivery.

Methods

This prospective observational study included 95 eligible pregnant women at a tertiary hospital over 18 months. All underwent transvaginal cervical length measurement between 36 and 36+6 weeks. Demographic and obstetric data (including Bishop score) were collected, and participants were followed until delivery. Outcomes were spontaneous labor by 39 weeks and the mode of delivery. Data were analyzed using chi-square tests for categorical variables and Pearson’s correlation for continuous variables, with significance at p < 0.05.

Results

Cervical length at 36-36+6 weeks showed no significant correlation with spontaneous labor by 39 weeks; all 95 women required induction. Longer cervical lengths were significantly associated with prolonged induction-to-delivery intervals (r = 0.491). Women with longer cervices more often required Foley catheter ripening (p = 0.001), while none with very short cervices (≤2.5 cm) required emergency cesarean delivery. Cervical length was not associated with gestational age at delivery (p = 0.065). These findings align with evidence that shorter cervical lengths predict faster inductions and higher vaginal delivery rates, whereas longer cervices are linked to the need for multiple induction methods and increased cesarean likelihood.

Conclusions

Cervical length measurement at 36-36+6 weeks can inform induction strategies at term. Although not predictive of spontaneous labor onset, longer cervical length was associated with prolonged induction, greater need for mechanical ripening, and higher cesarean risk. These results support incorporating cervical length assessment into preinduction evaluation. Identifying a longer cervix at 36 weeks can alert clinicians to the likely need for intensive induction methods (e.g., Foley catheter) and the potential for cesarean delivery if the cervix remains unfavorable. The findings align with guidelines advocating individualized induction based on objective cervical assessment. Combining the Bishop score with sonographic cervical length at 36-37 weeks may improve the prediction of induction success and guide delivery planning. Further research should refine how cervical length measurement can be integrated into standard induction protocols to improve maternal and neonatal outcomes.

## Introduction

Antenatal care encompasses a broad range of assessments aimed at ensuring maternal and fetal well-being throughout pregnancy. These include detailed history taking, physical examination, fetal heart monitoring, and ultrasonography. Ultrasonography has become indispensable in modern obstetric practice, providing essential information on fetal anatomy, growth patterns, placental location, and amniotic fluid volume. While first- and second-trimester scans are routinely performed for dating and anomaly detection, third-trimester scans are typically reserved for specific indications such as suspected fetal growth restriction, macrosomia, antepartum hemorrhage, or confirmation of fetal presentation [1].

As obstetric practice evolves, elective induction of labor at term has become increasingly common. This trend has been influenced by large clinical trials, such as the ARRIVE trial, which suggested that elective induction at 39 weeks may reduce cesarean section rates in selected populations [2]. With rising induction rates, there is a growing need for objective predictors of induction success. Traditionally, cervical favorability has been assessed through clinical examination, but this approach is limited by subjectivity and interobserver variability [3]. These limitations have prompted interest in more objective, reproducible methods of assessing cervical status.

Transvaginal ultrasound measurement of cervical length has emerged as a promising tool for evaluating cervical readiness in late pregnancy. Unlike clinical examination, ultrasound provides a quantifiable, operator-independent measurement that may better reflect cervical physiology. Several studies have explored the predictive value of third-trimester cervical length for labor progression and delivery outcomes. Pandis et al. reported that cervical length was a superior predictor of induction success compared with clinical assessment [3]. Giyahi et al. found that shorter cervical length at 36-38 weeks correlated with earlier labor, though not with mode of delivery [4]. Ramanathan et al. demonstrated that shorter cervical length at 37 weeks was associated with earlier delivery [5]. However, other studies have reported mixed results. Strobel et al. found no clear advantage of ultrasound over clinical assessment for predicting labor onset within 24-48 hours [6], while Vimercati et al. observed that women delivering post-term had significantly longer cervical lengths near term [7]. Maitra et al. identified cervical length as an independent predictor of induction success, with markedly higher cesarean rates when cervical length reached 4 cm [8]. Additional studies have reinforced the association between shorter cervical length and favorable induction outcomes [9,10], while Najam et al. suggested that serial measurements may be more predictive than a single late-pregnancy measurement [11].

Recent literature has expanded the understanding of cervical length dynamics across the third trimester. Tsakiridis et al. [12] highlighted the predictive value of third-trimester cervical length for late preterm birth, while Léhner et al. [13] demonstrated its association with the duration of the first stage of labor. Brieger et al. [14] described cervical changes across the third trimester, supporting the concept that cervical shortening is a gradual physiological process. Hughes et al. [15] confirmed cervical length as a strong prognostic marker for spontaneous preterm birth, while Hong et al. [16] showed that early third-trimester cervical shortening predicts preterm birth even in women with normal mid-trimester lengths.

Despite these insights, the predictive value of cervical length for spontaneous labor onset remains inconsistent across studies. Moreover, in settings where routine induction is practiced, spontaneous labor may not occur before induction, limiting the ability to evaluate cervical length as a predictor of natural labor onset. This was the case in the present study, where all participants underwent planned induction at 39 weeks. As a result, the study objective was reframed to evaluate the predictive value of cervical length for induction-related outcomes and mode of delivery.

Given the increasing use of planned induction and the need for objective predictors of induction behavior, this study evaluates whether cervical length measured at 36-36+6 weeks predicts induction-related outcomes and mode of delivery in a cohort where all participants underwent scheduled induction.

## Materials and methods

This prospective observational study was conducted over 18 months at Jubilee Mission Medical College and Research Institute, Thrissur, India. The study aimed to evaluate the predictive value of cervical length measured at 36-36+6 weeks for induction-related outcomes in term pregnancies undergoing planned induction. A sample size of 95 was calculated based on an expected correlation coefficient (r = 0.33, p < 0.001) between cervical length and labor outcomes from prior studies [4], with a 95% confidence level and 90% power.

Study population

Eligible participants were pregnant women with a singleton fetus in cephalic presentation at 36-36+6 weeks’ gestation. Gestational age was confirmed by the last menstrual period or first-trimester ultrasound. Exclusion criteria included previous cesarean section or uterine surgery, major fetal anomalies, hypertensive disorders of pregnancy, fetal growth restriction, contraindications to prostaglandin E2, prelabor rupture of membranes, maternal infection or fever ≥38°C, and contraindications to induction such as placenta previa or transverse lie.

Data collection

After informed consent, demographic and obstetric data were collected using a structured pro forma. A transvaginal ultrasound was used to measure cervical length at 36-36+6 weeks. The ultrasound was performed with the patient in the lithotomy position using a high-frequency transvaginal probe. Care was taken to avoid undue pressure on the cervix, which could artificially shorten the measurement. The shortest of the three measurements was recorded as the final cervical length.

Participants were followed until delivery. Induction methods, induction-to-active labor interval, total induction-to-delivery duration, and mode of delivery were recorded. Induction methods included prostaglandin E2 gel, Foley catheter insertion, amniotomy, and oxytocin infusion. The choice of induction method was based on clinical judgment and institutional protocols.

Outcome measures

Primary outcomes included induction-to-active labor interval, total induction-to-delivery interval, need for cervical ripening methods, and mode of delivery. Secondary outcomes included gestational age at delivery and use of specific induction methods.

Statistical analysis

Data were analyzed using IBM SPSS Statistics for Windows, version 27.0 (released 2019; IBM Corp., Armonk, NY, USA). Continuous variables were summarized as mean ± SD; categorical variables as frequencies and percentages. Normality of continuous variables was assessed using the Kolmogorov-Smirnov test. Chi-square tests and Fisher’s exact test were used for categorical comparisons. Pearson’s correlation assessed relationships between cervical length and continuous outcomes. A p-value < 0.05 was considered statistically significant.

## Results

Ninety-five women underwent induction at term. The mean maternal age was in the late twenties, with the majority of participants falling into the 26- to 30-year age group. Primigravidae comprised 62% of the study population, while multigravidae accounted for 38%. Vaginal delivery occurred in 79% of cases, including 62% spontaneous vaginal deliveries and 17% assisted vaginal deliveries. Emergency cesarean section was required in 21% of cases, primarily for non-progression of labor.

No participant experienced spontaneous labor before 39 weeks, precluding analysis of cervical length as a predictor of spontaneous labor onset. Subsequent analyses focused on induction outcomes.

Cervical length and mode of delivery

None of the women with the shortest cervices (≤2.5 cm) required an emergency cesarean section. Cesarean rates increased progressively with increasing cervical length. Among women with cervical length >3.5 cm, 53% required cesarean delivery. This suggests that longer cervical length at 36-36+6 weeks is associated with a higher likelihood of operative delivery following induction.

Cervical length and induction-to-delivery interval

Longer cervical length was significantly associated with prolonged induction-to-delivery intervals (χ² = 7.896, p = 0.019). Women with a cervical length >3.5 cm were more likely to experience induction-to-delivery intervals exceeding 15 hours, while those with shorter cervices were more likely to deliver within 10-14 hours.

Induction methods

Use of a Foley catheter was significantly associated with longer cervical length. All women with cervical length >3.5 cm required Foley catheter insertion, while none of the women with cervical length ≤2.5 cm required mechanical ripening. Prostaglandin E2, amniotomy, and oxytocin use did not differ significantly across cervical length groups, reflecting routine labor ward practice.

Gestational age at delivery

Cervical length was not significantly associated with gestational age at delivery (p = 0.097). Most women delivered between 38 and 39 weeks, regardless of cervical length. Table 1 and Table 2 present detailed demographic, obstetric, and induction-related findings.

**Table 1:** Sociodemographic and obstetric characteristics of study participants (N = 95) FTND, full-term normal delivery; LSCS, lower segment cesarean section; RMLE, right mediolateral episiotomy

Category	Count	Percentage (%)
Age in years (N = 95)		
≤20	2	2
21-25	26	27
26-30	48	51
31-35	14	15
≥36	5	5
Mode of delivery (N = 95)		
Emergency LSCS	19	21
FT vacuum-assisted with RMLE	17	17
FTND with RMLE	59	62
Gravidity score (N = 95)		
Primi	59	62
Multi	36	38
Cervical length (in cm) (N = 95)		
Below 3.5	60	63
Above 3.5	35	37

**Table 2:** Distribution of induction-to-delivery interval, induction methods, and gestational age by cervical length among study participants A p-value < 0.05 was considered statistically significant. PGE₂, prostaglandin E₂

Outcome		Cervical length		Significance
		Below 3.5	Above 3.5	
Induction-to-delivery interval (hours) (N = 76)	<9	8 (15.7%)	1 (4.0%)	χ² = 7.896, p = 0.019, statistically significant
	10-14	32 (62.7%)	11 (44.0%)	
	>15	11 (21.6%)	13 (52.0%)	
Combination of induction methods (N = 95)	Only oxytocin	5 (8.3%)	0 (0.0%)	Fisher’s exact test, p = 0.128, not statistically significant
	Only PGE₂	2 (3.3%)	0 (0.0%)	
	Only Foley	4 (6.7%)	2 (5.7%)	
	Foley and oxytocin	4 (6.7%)	6 (17.1%)	
	Foley and PGE₂	4 (6.7%)	3 (8.6%)	
	Foley, PGE₂, oxytocin	41 (68.3%)	24 (68.6%)	
Gestational age at delivery (N = 95)	37 weeks	10 (16.7%)	2 (5.7%)	χ² = 4.660, p = 0.097, not statistically significant
	38 weeks	22 (36.7%)	20 (57.1%)	
	39 weeks	28 (46.7%)	13 (37.1%)	

## Discussion

This study evaluated the predictive value of cervical length measured at 36-36+6 weeks in a cohort where all women underwent planned induction. Because no participant entered spontaneous labor, cervical length could not be assessed as a predictor of spontaneous labor onset. Instead, the analysis focused on induction outcomes.

Shorter cervical length was associated with shorter induction-to-delivery intervals, reduced need for mechanical ripening, and higher rates of vaginal delivery. These findings align with previous studies demonstrating that cervical length is a meaningful predictor of induction behavior [3,8]. The association between shorter cervical length and favorable induction outcomes may reflect underlying cervical physiology, as shorter cervices may be more responsive to induction agents.

The strong association between longer cervical length and Foley catheter use reflects standard clinical practice, where mechanical ripening is preferred for unripe cervices. Other induction methods were used broadly across cervical length groups, consistent with routine labor ward protocols. The lack of a significant association between cervical length and prostaglandin E₂, amniotomy, or oxytocin use suggests that these methods are applied based on clinical judgment rather than cervical length alone.

Cervical length was not associated with gestational age at delivery, consistent with studies reporting limited predictive value of late third-trimester cervical length for spontaneous labor onset [6,7]. This may reflect the influence of routine induction practices, which override natural labor patterns.

A key limitation of this study is the absence of spontaneous labor cases, which restricts conclusions regarding natural labor onset. However, this reflects real-world practice in settings with routine induction by 39 weeks. The study also did not include Bishop score assessment, so findings reflect the predictive value of cervical length alone.

Despite these limitations, the study provides valuable insights into the role of cervical length in predicting induction outcomes. The findings support the use of late-pregnancy cervical length assessment as a practical tool for anticipating induction complexity and guiding individualized counseling and management strategies.

## Conclusions

Cervical length measured at 36-36+6 weeks demonstrated meaningful predictive value for induction-related outcomes in a cohort where all women underwent planned induction. Shorter cervical length was associated with reduced need for cervical ripening interventions, shorter induction-to-delivery intervals, and a higher likelihood of vaginal delivery. Longer cervical length correlated with prolonged induction duration and increased likelihood of cesarean delivery.

These findings support the use of late-pregnancy cervical length assessment as a practical tool for anticipating induction complexity and guiding individualized counseling and management strategies. In clinical settings where routine induction is common, cervical length may serve as an important adjunct in optimizing induction planning and improving patient preparedness. Further research in diverse populations may help refine its predictive role across broader obstetric contexts.

## References

[1] Williams JW (2023). Williams Obstetrics, 26th Edition. AccessMedicine.

[2] Grobman WA, Rice MM, Reddy UM (2018). Labor induction versus expectant management in low-risk nulliparous women. N Engl J Med.

[3] Pandis GK, Papageorghiou AT, Ramanathan VG, Thompson MO, Nicolaides KH (2001). Preinduction sonographic measurement of cervical length in the prediction of successful induction of labor. Ultrasound Obstet Gynecol.

[4] Giyahi H, Marsosi V, Faghihzadeh S, Kalbasi M, Lamyian M (2018). Sonographic measurement of cervical length and its relation to the onset of spontaneous labour and the mode of delivery. Natl Med J India.

[5] Ramanathan G, Yu C, Osei E, Nicolaides KH (2003). Ultrasound examination at 37 weeks' gestation in the prediction of pregnancy outcome: the value of cervical assessment. Ultrasound Obstet Gynecol.

[6] Strobel E, Sladkevicius P, Rovas L, De Smet F, Karlsson ED, Valentin L (2006). Bishop score and ultrasound assessment of the cervix for prediction of time to onset of labor and time to delivery in prolonged pregnancy. Ultrasound Obstet Gynecol.

[7] Vimercati A, Greco P, Lopalco P (2001). The value of ultrasonographic examination of the uterine cervix in predicting post-term pregnancy. J Perinat Med.

[8] Maitra N, Sharma D, Agarwal S (2009). Transvaginal measurement of cervical length in the prediction of successful induction of labour. J Obstet Gynaecol.

[9] Vankayalapati P, Sethna F, Roberts N, Ngeh N, Thilaganathan B, Bhide A (2008). Ultrasound assessment of cervical length in prolonged pregnancy: prediction of spontaneous onset of labor and successful vaginal delivery. Ultrasound Obstet Gynecol.

[10] Souka AP, Papastefanou I, Papadopoulos G, Chrelias C, Kassanos D (2015). Cervical length in late second and third trimesters: a mixture model for predicting delivery. Ultrasound Obstet Gynecol.

[11] Najam S, Ishaq S, Anjum S, Hassan S (2020). Role of cervical length measurement at 37 weeks in prediction of time of delivery. Int J Health Sci Res.

[12] Tsakiridis I, Dagklis T, Sotiriadis A, Mamopoulos A, Zepiridis L, Athanasiadis A (2023). Third-trimester cervical length assessment for the prediction of spontaneous late preterm birth. J Matern Fetal Neonatal Med.

[13] Léhner G, Reif P, Avian A, Kollmann M, Lakovschek IC, Lang U, Ulrich D (2019). Does third trimester cervical length predict duration of first stage of labor?. Wien Klin Wochenschr.

[14] Brieger GM, Ning XH, Dawkins RR, Ying KQ, Weng C, Chang AM, Haines CJ (1997). Transvaginal sonographic assessment of cervical dynamics during the third trimester of normal pregnancy. Acta Obstet Gynecol Scand.

[15] Hughes K, Ford H, Thangaratinam S, Brennecke S, Mol BW, Wang R (2023). Diagnosis or prognosis? An umbrella review of mid-trimester cervical length and spontaneous preterm birth. BJOG.

[16] Hong S, Lee SU, Won S, Kang BS, Kim O, Park IY, Ko HS (2024). Predictive value of short cervix in early third trimester for preterm birth in women with normal mid-trimester cervical length. Am J Obstet Gynecol MFM.

